# Impact of Pre-operative Embolization With Onyx for Brain Arteriovenous Malformation Surgery

**DOI:** 10.3389/fneur.2022.875260

**Published:** 2022-04-26

**Authors:** Tsuyoshi Izumo, Kazuaki Okamura, Ryotaro Takahira, Yuki Matsunaga, Eisaku Sadakata, Hajime Maeda, Susumu Yamaguchi, Shiro Baba, Yoichi Morofuji, Takeshi Hiu, Nobutaka Horie, Takeo Anda, Naoki Kitagawa, Yoshiharu Tokunaga, Kentaro Hayashi, Yasushi Matsumoto, Izumi Nagata, Takayuki Matsuo

**Affiliations:** ^1^Department of Neurosurgery, Nagasaki University Graduate School of Biomedical Sciences, Nagasaki, Japan; ^2^Department of Neurosurgery, Nagasaki Rosai Hospital, Nagasaki, Japan; ^3^Department of Neurosurgery, Nagasaki Prefecture Shimabara Hospital, Nagasaki, Japan; ^4^Advanced Stroke Center, Shimane University Hospital, Izumo, Japan; ^5^Department of Neuroendovascular Therapy, Kohnan Hospital, Sendai, Japan; ^6^Department of Neurosurgery, Kokura Memorial Hospital, Kitakyushu, Japan

**Keywords:** Onyx, *N*-butyl cyanoacrylate, coils, neurosurgery, cerebral arteriovenous malformation (cAVM)

## Abstract

**Objective:**

To clarify the safety and efficacy of pre-operative embolization using Onyx liquid embolic agent (Onyx; ev3) compared with *N*-butyl cyanoacrylate (NBCA; Cordis Neurovascular, Inc.) or coils in cerebral arteriovenous malformation (AVM) surgery.

**Methods:**

This was a retrospective review of a prospectively collected clinical database of brain AVMs treated at our institute from January 2005 to March 2021. A total of 38 consecutive patients who underwent AVM resection after pre-operative embolization were included. Based on pre-operative embolization materials, the patients were divided into the pre-Onyx group (*n* = 16), in which NBCA or coils were used for embolization, and the Onyx group (*n* = 22). Patient characteristics and treatment results were compared between the two groups.

**Results:**

Patient characteristics were comparable between the two groups in terms of age, sex, and rupture status. While the Spetzler–Martin grade was also similar between the two groups, the location of the AVM nidus in the eloquent area was slightly higher in patients in the Onyx group (72.7%) than in patients in the pre-Onyx group (43.8%) (*P* = 0.09). The embolization rate was higher in the pre-Onyx group (mean: 63.0%; range: 12.7–100%) than in the Onyx group (mean: 50.0%; range: 15.8–100%), but the difference was not statistically significant (*P* = 0.06). The time needed for surgical removal was shorter in the Onyx group (mean: 354.8 min; range: 144–884 min) than in the pre-Onyx group (mean: 457.9 min; range: 240–1,294 min); however, this difference was not statistically significant (*P* = 0.13). The amount of intraoperative bleeding was significantly lower in the Onyx group (mean: 129.8 ml; range: 20–540 mL) than in the pre-Onyx group (mean: 448.8 mL; range: 120–1,550 ml) (*P* = 0.0008). The surgical complication rates were comparable between the two groups (pre-Onyx group, 18.8%; Onyx group, 4.5%; *P* = 0.29).

**Conclusions:**

Pre-operative embolization with Onyx can significantly reduce the amount of intraoperative bleeding in AVM resection and may contribute to safe AVM surgery.

## Introduction

Pre-operative endovascular embolization for brain arteriovenous malformation (AVM) has been widely performed in recent years, and good treatment results have been reported (1–3). Various materials have been conventionally used for embolization of AVMs (4–6). In Japan, *N*-butyl cyanoacrylate (NBCA; Cordis Neurovascular, Inc., Miami, FL) has been widely used for pre-operative embolization of AVMs, but after the approval of the Onyx liquid embolic system (Onyx; ev3, CA, USA) by the Japanese Ministry of Health, Labor and Welfare in 2008, this embolic material is being increasingly used in the treatment of AVMs. Compared with NBCA, Onyx has slower cast formation, flow-independent delivery, cohesiveness, and smaller minimum diameter of embolized vessel (7–9). These features enable the usage of the plug and push method and penetration into smaller microvasculature, leading to nidus occlusion. For these reasons, pre-operative embolization for AVM surgery using Onyx is expected to be more effective than using NBCA (10, 11). According to a review of 1,042 AVM cases from Japan that underwent embolization, coils were used in 165 embolization procedures (15.8%); NBCA in 627 (60.2%); and Onyx in 432 (41.5%) (11). However, a multicenter randomized controlled trial and some observational studies that directly compared Onyx and NBCA failed to show that pre-operative embolization using Onyx resulted in superior surgical outcomes (1, 8, 12).

The purpose of this study was to investigate the safety and efficacy of pre-operative embolization using Onyx by comparing it with coils or NBCA in cerebral AVM surgery using real-world data.

## Materials and Methods

### Study Population and Data Analysis

This study was carried out in accordance with the tenets of the Declaration of Helsinki and was approved by the Institutional Review Board of Nagasaki University Hospital (No. 21081606). Written informed consent was obtained from all participants.

Between January 2005 and March 2021, 38 consecutive patients with AVMs who were treated at our hospital with endovascular embolization and microsurgical resection were included in this study. The data were prospectively maintained in the AVM database. All embolization procedures were performed by two board-certified neuroendovascular therapists (H.K. and N.H.) of the Japan Neuroendovascular Therapy Society. All surgeries were performed by two board-certified neurosurgeons (I.N. and T.I.) of the Japan Neurosurgical Society.

Onyx became available at our institution on May 1, 2014; since then, we have changed the first choice of embolization materials from NBCA to Onyx for the pre-operative embolization of brain AVMs. Our basic policy was to aim for nidus embolization using the plug and push method with Onyx embolization. Thus, the patients included in this study were divided into pre-Onyx and Onyx groups, depending on the embolization material. The prospectively collected clinical data were reviewed and analyzed by a single neurosurgeon (T.I.). The following data were included: age, sex, symptoms at presentation, Spetzler–Martin grade, rupture status, embolic material, number of embolized feeding arteries, number of embolization sessions, embolization rate, endovascular procedure complications, operative time, intraoperative blood loss, obliteration rate after surgery, surgical complications, and pre-operative and post-operative (at discharge) modified Rankin Scale (mRS) score.

The pre-embolized and post-embolized nidus of the AVM was manually traced in each of the anterior-posterior (AP) and lateral (LAT) views in the digital subtraction angiogram (DSA) image using Synapse Vincent software (Fujifilm, Japan), and the area was automatically calculated (mm^2^). The embolization rate was calculated for each AP and LAT view and is described as the average value. After surgery, the obliteration rate was defined utilizing post-operative DSA performed after 10 days. A good neurological outcome was defined as an mRS score <3, and a poor outcome was defined as an mRS score ≥3.

### Statistical Methods

Statistical analyses were performed to compare the two patient groups. Continuous variables are presented as the mean and standard deviation (SD) and as the median and range, while categorical variables are presented as frequencies. Statistical analysis was conducted using the Student's *t*-test or Mann–Whitney *U*-test for continuous variables and Fisher's exact test for categorical variables, as appropriate. All statistical analyses were performed using SPSS for Windows (version 24.0; IBM, Armonk, NY, USA). Differences were considered statistically significant at *P* < 0.05.

## Results

### Baseline Characteristics of Participants

There were 16 patients in the pre-Onyx group and 22 in the Onyx group. Patient characteristics at baseline were similar between the two groups (Table 1). The mean age was 40.9 ± 20.3 years in the pre-Onyx group and 41.4 ± 22.9 years in the Onyx group (*P* = 0.95). The proportion of female patients was 25.0% (4/16) in the pre-Onyx group and 45.5% (10/22) in the Onyx group (*P* = 0.31). The most frequent symptom at presentation was disturbance of consciousness in both groups. Seizure was observed slightly more frequently in the pre-Onyx group. One patient in the Onyx group was asymptomatic.

**Table 1:** Patient demographics.

	**Pre-onyx group**	**Onyx group**	***P-*value**
No. of patients	16	22	
**Age, years**
Mean ± SD	40.9 ± 20.3	41.4 ± 22.9	0.95
Median (range)	49.5 (8–67)	46 (7–81)	
**Sex (%)**
Female	4 (25)	10 (45.5)	0.31
Male	12 (75)	12 (54.5)	
**Presenting symptoms**
Consciousness disturbance	7	10	
Hemiparesis	4	9	
Sensory disturbance	0	1	
Seizure	4	1	
Headache	6	6	
Nausea/vomiting	5	2	
Visual field defect	2	2	
Vertigo	1	1	
None	0	1	

*SD, standard deviation*.

### Baseline Characteristics of AVMs

Baseline AVM characteristics were comparable between the two groups (Table 2). In the pre-Onyx group the AVMs most frequently in the right side, while in the Onyx group the AVMs were located equally in the right and left side; however, this difference was not significant (*P* = 0.64). There were no significant difference in Spetzler-Martin grades between the Onyx and pre-Onyx grous (*p* = 0.32). Conversely, there were increasing trends of patients with the AVM nidus in the eloquent area in the Onyx group (72.7%) compared with those in the pre-Onyx group (43.8%) (*P* = 0.09). The proportion of patients presenting with AVM rupture was 87.5% in the pre-Onyx group and 91% in the Onyx group (*P* = 1.00). A good pretreatment mRS score (0–2) was found in 62.5% of patients in the pre-Onyx group and 50% in the Onyx group (*P* = 0.52).

**Table 2:** AVM characteristics.

	**Number (%)**		
	**Pre-onyx group**	**Onyx group**	***P*-value**
No. of patients	16	22	
**AVM location**			0.64
Left	6 (37.5)	10 (45.5)	
Right	8 (50)	10 (45.5)	
Midline	2 (12.5)	2 (9.0)	
**Spetzler-Martin grade**			0.32^*^
I	4 (25)	3 (13.6)	
II	6 (37.5)	6 (27.3)	
III	5 (31.3)	10 (45.5)	
IV	1 (5.2)	3 (13.6)	
**AVM size, maximum diameter**			0.34
<3 cm	10 (62.5)	10 (45.5)	
3–6 cm	6 (37.5)	11 (50)	
>3 cm	0 (0)	1 (4.5)	
Nidus in eloquent area	7 (43.8)	16 (72.7)	0.09
Deep venous drainage	6 (37.5)	6 (27.3)	0.72
**Rupture status**			1.00
Ruptured	14 (87.5)	20 (91.0)	
Unruptured	2 (12.5)	2 (9.0)	
**Preop mRS score**			0.52
0–2	10 (62.5)	11 (50)	
3–5	6 (37.5)	11 (50)	

* *I + II/III + IV*.

*AVM, arteriovenous malformation; mRS, modified Rankin Scale*.

### Embolization Results

In the pre-Onyx group, NBCA alone was used in seven patients, coil (the Guglielmi detachable coil; Stryker Neurovascular, MI, USA or ED coil, Kaneka Medix, Japan) alone was used in four patients, and a combination of NBCA and coil was used in five patients (Table 3). In the Onyx group, Onyx alone was used in 21 patients and a combination of Onyx and NBCA was used in one patient. The mean number of embolized arteries was 1.94 ± 0.33 in the pre-Onyx group and 2.27 ± 0.28 in the Onyx group (*P* = 0.45) (Table 4). The mean number of embolization sessions was 1.06 ± 0.069 in the pre-Onyx group and 1.09 ± 0.059 in the Onyx group (*P* = 0.76). The mean embolization rate was lower in the Onyx group (50.0 ± 4.43%) than in the pre-Onyx group (63.0 ± 5.20%), but the difference was not significant (*P* = 0.06). Pre-operative embolization complications occurred in two patients (both in the Onyx group). One patient experienced transient aphasia due to cerebral infarction caused by migration of Onyx to a passing artery, and one patient experienced a stuck microcatheter. Thus, the complication rate was 0% in the pre-Onyx group and 9.1% in the Onyx group; the difference was not statistically significant (*P* = 0.50).

**Table 3:** Embolization materials.

	**Pre-onyx group**		**Onyx group**	
	NBCA	7	Onyx	21
	coils	5	Onyx + NBCA	1
	NBCA + coils	4		
Total		16		22

*NBCA, N-butyl cyanoacrylate*.

**Table 4:** Treatment results.

	**Pre-Onyx group**	**Onyx group**	***P-*value**
No. of patients	16	22	
**Number of embolized arteries**
Mean ± SD	1.94 ± 0.33	2.27 ± 0.28	0.45
Median (range)	2 (1–3)	2 (1–4)	
**Number of embolization sessions**
Mean ± SD	1.06 ± 0.069	1.09 ± 0.059	0.76
Median (range)	1 (1–2)	1 (1–2)	
**Embolization rate (%)**
Mean ± SD	63.0 ± 5.20	50.0 ± 4.43	0.06
Median (range)	62.0 (12.7–100)	52.6 (15.8–100)	
**Complications of embolization**
No. (%)	0 (0)	2 (9.1)	0.50
**Operative time (minutes)**
Mean ± SD	457.9 ± 50.4	354.8 ± 43.0	0.13
Median (range)	379 (240–1,294)	311 (144–884)	
**Intraoperative bleeding (ml)**
Mean ± SD	448.8 ± 388.6	129.8 ± 117.0	0.0008
Median (range)	300 (120–1,550)	97 (20–540)	
Total removal no. (%)	16 (100)	22 (100)	1.00
**Surgical complication**
No. (%)	3 (18.8)	1 (4.5)	0.29
**Post-operative mRS score**			1.00
0–2	11 (68.8)	15 (68.4)	
3–5	5 (31.2)	7 (31.6)	

*SD, standard deviation; mRS, modified Rankin Scale*.

### Surgical Results

The mean operative time was comparable between the two groups (457.9 ± 50.4 min in the pre-Onyx group and 354.8 ± 43.0 min in the Onyx group, *P* = 0.13) (Table 4). On the other hand, mean intraoperative bleeding was significantly less in the Onyx group (129.8 ± 117.0 mL) than in the pre-Onyx group (448.8 ± 388.6 mL) (*P* = 0.0008). Total removal was achieved in all patients in both groups. Surgical complications occurred in three patients in the pre-Onyx group and one patient in the Onyx group. In the pre-Onyx group, symptomatic hemorrhage occurred in two patients. Among them, one patient underwent re-craniotomy and hematoma removal, and one patient underwent ventricular drainage. The other patient experienced wound infection. In the Onyx group, post-operative intracerebral hemorrhage occurred in one patient who underwent re-craniotomy and hematoma removal. Thus, the surgical complication rate was 18.8% in the pre-Onyx group and 4.5% in the Onyx group (*P* = 0.29). No post-operative worsening of the mRS was observed in either group. A good post-operative outcome (mRS 0–2) was achieved in 68.8% (11/16) of the pre-Onyx group and 68.4% (15/22) of the Onyx group (*P* = 1.00).

### Representative Cases Presentation

**Figure 1:**
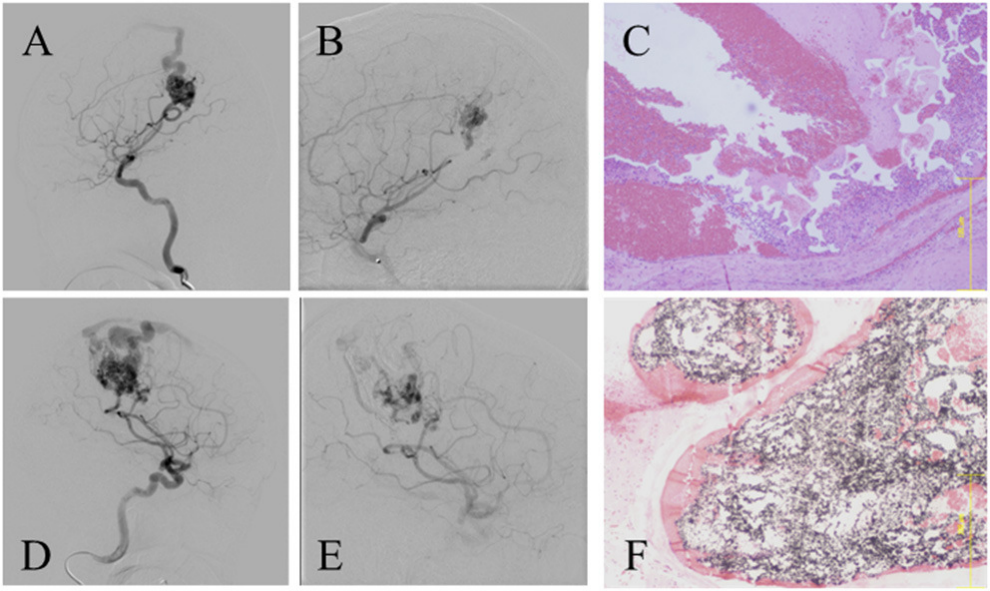
**(A,B)** Lateral projection digital subtraction angiogram (DSA) showing Spetzler-Martin grade 1 AVM pre-embolization and post embolization using NBCA, respectively. The calculated embolization rate was 68.8%. **(C)** pathological findings of resected angiographically disappeared nidus showing remarkable red blood cells in the microvasculatures (Hematoxylin-Eosin staining, ×25). **(D,E)** Lateral projection DSA showing Spetzler-Martin grade 3 AVM pre-embolization and post embolization with Onyx, respectively. The calculated embolization rate was 52.1%. **(F)** Pathological findings of resected angiographically disappeared nidus showing the Onyx cast without red blood cells in the microvasculatures (Hematoxylin-Eosin staining, ×25). AVM, arteriovenous malformation; NBCA, *N*-butyl cyanoacrylate.

We compared the post-operative histopathological findings between patients who underwent NBCA pre-operative embolization (Figures 1A–C) and Onyx (Figures 1D–F). The first case was a Spetzler–Martin grade 1 right parietal unruptured AVM. The patient underwent pre-operative embolization with NBCA, and the embolization rate was 68.8%. The post-operative pathological findings of the NBCA-embolized nidus revealed that there were significant red blood cells (RBCs) in some areas of the removed nidus. This area had disappeared on cerebral angiography after the NBCA-embolization. The second case was a Spetzler–Martin grade 3 ruptured AVM. The patient underwent pre-operative embolization with Onyx, and the embolization rate was 52.1%. The post-operative pathological findings of the angiographically disappeared area of the nidus showed the Onyx cast in the AVM microvasculature and sparse RBCs.

## Discussion

In this study, pre-operative embolization with Onyx significantly reduced bleeding in AVM surgery compared with NBCA or coil embolization. In addition, the operation time tended to be shorter in the Onyx group, although the difference was not significant. The Onyx group tended to have more lesions in the eloquent area; however, no cases with worsened functional status were observed after surgery, and the same treatment prognosis as in the pre-Onyx group could be achieved.

Intraoperative bleeding is a significant risk factor for surgical complications after surgery for cerebral AVMs. Therefore, it is crucial to suppress the bleeding to a small amount using a precise maneuver (13–17). Wong et al. studied 977 surgical cases of cerebral AVM and reported that early neurological sequelae and permanent complications were observed in 9.3 and 3.4% of patients, respectively (18). Multivariate analysis revealed that two or more units of red blood cell transfusions or 1,000 mL or more of intraoperative bleeding were significant risk factors for complications (18). Thus, performing surgery for AVMs with a small amount of intraoperative bleeding contributes to improved prognosis for post-operative patients.

The usefulness of pre-operative embolization to reduce intraoperative bleeding in brain AVM surgery has been widely reported. Spetzler et al. reported the effectiveness of surgical removal following multiple pre-operative embolizations for Spetzler–Martin high-grade classification of brain AVMs (19). As a result, pre-operative embolization shortened the surgical time and reduced the amount of bleeding, leading to a reduction in surgical complications and improved long-term neurological outcomes. DeMeritt et al. reported that 70% of patients in the pre-operative embolization with surgery group had no signs of neurological loss 1 week after surgery, compared with 41% (*P* < 0.05) in the surgery alone group (20). Thus, pre-operative embolization seems to be an essential procedure for improving the safety of surgery for brain AVMs (21).

Conventionally, NBCA has been widely used as an embolic substance for endovascular embolization. Several reports have shown that the effect of embolization with NBCA on brain AVMs is beneficial. Meisel et al. investigated the impact of partial embolization on brain AVMs using NBCA and reported a 31% reduction in the annual bleeding rate compared to pretreatment (22). Moreover, Wikholm et al. followed up 150 cases of cerebral arteriovenous malformation that underwent embolization with NBCA for an average of 6.3 years and reported a high occlusion rate of at least 90% and stable neurological signs (23).

On the other hand, pre-operative embolization using NBCA requires obliteration of at least two-thirds or more volume of the nidus to be effective in brain AVM surgery (24). Multiple embolization sessions are mandatory when using NBCA, especially for large brain AVMs, to achieve adequate nidus obliteration in radiation exposure and hemodynamic safety. However, some reports have shown that an increase in the number of treatment sessions is a significant factor in the post-operative neurological complication rate, and aggressive treatment using NBCA should be modestly applied to select cases (2, 25). NBCA has been used for a long time; however, medical professionals have waited a long time for the emergence of an embolic substance with greater maneuverability and better safety to replace it.

To overcome this, a new liquid embolic substance, the Onyx liquid embolic system, has been developed and launched on the market. Compared to the NBCA, Onyx has (1) slower cast formation, (2) blood flow-independent delivery, (3) cohesiveness, and (4) allows for penetration into a smaller vessel size (7–9). These characteristics enable the plug-and-push method and embolization of the brain AVM microvasculature. Several observational studies have reported that pre-operative embolization with Onyx may improve surgical outcomes for brain AVMs (9, 10, 26–28). On the other hand, a multicenter randomized controlled trial and some observational studies that directly compared Onyx and NBCA failed to show that pre-operative embolization using Onyx resulted in superior surgical outcomes (1, 8, 12). Thus, the effectiveness of pre-operative embolization with Onyx in AVM surgery is unclear.

In the present study, pre-operative embolization with Onyx significantly reduced intraoperative bleeding during AVM surgery compared to NBCA or coils. In addition, although the difference was not significant, the operation time tended to be shorter when using Onyx. We believe that the nidus occlusion technique makes the most of the unique characteristics of Onyx, which made it possible to achieve these results. As shown in Figure 1, when pre-operative embolization using NBCA was performed, even if the blood flow of the nidus appeared to disappear on cerebral angiography, the histopathological image revealed prominent RBCs in the nidus. This phenomenon is a result of the NBCA characteristics, which make it difficult to reach the microvessels of the nidus. In other words, NBCA embolization results in feeder occlusion, and blood flow from different routes results in persistent blood flow into the nidus.

On the other hand, the pathological findings in the Onyx-embolized nidus revealed Onyx cast in the microvessels instead of the erythrocytes, which result from the characteristic of Onyx to reach the microvasculature by the plug-and-push method: its ability to continuously inject and fill the nidus after the formation of the plug. These differences in the penetration of Onyx and NBCA into microvessels were the leading cause of the significant reduction in intraoperative bleeding in the Onyx group compared to the pre-Onyx group in this study. In other words, the nidus occlusion policy with the plug-and-push method is crucial for achieving a significant reduction in intraoperative bleeding during AVM surgery *via* pre-operative embolization with Onyx.

The morbidity and mortality rates of the combined strategy, which included Onyx pre-operative embolization and surgery, were reported to be 0–21% and 0–2%, respectively (8, 10, 12, 26, 29). In the present study, the post-operative morbidity rate was 4.5%, and mortality did not occur, indicating good treatment results equivalent to those in the aforementioned studies. Moreover, although the Onyx group had a more eloquent location nidus than the pre-Onyx group, the post-operative prognosis was similar. There were no cases showing deterioration of the mRS score in the Onyx group. As mentioned above, accurate dissection around the nidus in a bloodless field is crucial for safe AVM surgery, especially for eloquent lesions (13–15, 17, 18, 30). We believe that our plug-and-push method with Onyx pre-operative embolization contributed to the achievement of an excellent post-operative prognosis due to reduced intraoperative bleeding because of the nidus occlusion policy.

Some of the limitations of this study were (1) the small sample size, (2) retrospective analysis, (3) patients recruited from a few centers, (4) absence of randomization, and (5) outcome assessment by neurosurgeons at our institutes, without external validation. The sample size was also insufficient for subgroup analysis based on the Spetzler–Martin grade of the AVMs and/or other characteristics. Moreover, two neurosurgeons (T.I. and N.I.) operated on all the patients in this series. Thus, care should be taken when generalizing our results to patients with AVM.

Although further studies will be needed to conclusively demonstrate the impact of Onyx pre-operative embolization for AVM surgery, this is the first study to show that presurgical embolization using Onyx results in significantly less intraoperative blood loss and a marginally shorter operation time compared with the pre-Onyx era, with favorable post-treatment outcomes.

## Conclusions

Pre-operative embolization with Onyx could significantly reduce the amount of intraoperative bleeding in AVM resection and marginally shorten the operative time compared with the pre-Onyx era. The favorable treatment outcomes of the Onyx group were comparable with those of previous reports. We believe that these results could be achieved by our nidus occlusion policy using the plug-and-push method with Onyx. Pre-operative embolization using Onyx may contribute to safe AVM surgery.

## Data Availability Statement

The raw data supporting the conclusions of this article will be made available by the authors, without undue reservation.

## Ethics Statement

The studies involving human participants were reviewed and approved by the Institutional Review Board of Nagasaki University Hospital. Written informed consent to participate in this study was provided by the participants' legal guardian/next of kin.

## Author Contributions

TI: conceptualization, methodology, visualization, and writing—original draft preparation. TI, KO, RT, YMatsun, ES, and HM: data curation. TI, NH, and KH: formal analysis. TI and TM: funding acquisition. TI and YMo: investigation. TI, IN, and TM: project administration. YMatsum, IN, and TM: supervision. NK and YT: validation. TI, SY, SB, TH, and TA: writing—review and editing. All authors have read and agreed to the published version of the manuscript.

## Funding

This study was funded by Grants-in-Aid for Scientific Research (C) 18K08973 (to TI), (C) 21K09180 (to TI), and (C) 21K09129 (to TM).

## Conflict of Interest

The authors declare that the research was conducted in the absence of any commercial or financial relationships that could be construed as a potential conflict of interest.

## Publisher's Note

All claims expressed in this article are solely those of the authors and do not necessarily represent those of their affiliated organizations, or those of the publisher, the editors and the reviewers. Any product that may be evaluated in this article, or claim that may be made by its manufacturer, is not guaranteed or endorsed by the publisher.
